# Clinical and Molecular Characterization of KRAS-Mutated Renal Cell Carcinoma

**DOI:** 10.3390/cancers17233832

**Published:** 2025-11-29

**Authors:** Andrea Lopez Sanmiguel, Yash S. Khandwala, Kuo Fengshen, Mark Dawidek, Ethan Tse, Daniel Barbakoff, Lina Posada Calderon, Maria I. Carlo, Jonathan Coleman, Paul Russo, Satish K. Tickoo, Victor E. Reuter, Ed Reznik, Ying-Bei Chen, A. Ari Hakimi

**Affiliations:** 1 Department of Surgery, Urology Service, Memorial Sloan Kettering Cancer Center, New York, NY 10065, USA; lopeza14@mskcc.org (A.L.S.); khandwy1@mskcc.org (Y.S.K.); kuof@mskcc.org (K.F.); barbakd1@mskcc.org (D.B.); lip2019@nyp.org (L.P.C.); colemanj@mskcc.org (J.C.); russop@mskcc.org (P.R.); 2 Immunogenomics and Precision Oncology Platform, Memorial Sloan Kettering Cancer Center, New York, NY 10065, USA; 3 Department of Urologic Sciences, Faculty of Medicine, The University of British Columbia, Vancouver, BC V5Z 1M9, Canada; 4 Department of Epidemiology and Biostatistics, Memorial Sloan Kettering Cancer Center, New York, NY 10065, USA; tsee@mskcc.org (E.T.); reznike@mskcc.org (E.R.); 5 Department of Urology, New York Presbyterian—Weill Cornell Medicine, New York, NY 10065, USA; 6 Genitourinary Medical Oncology Service, Memorial Sloan Kettering Cancer Center, New York, NY 10065, USA; carlom@mskcc.org; 7 Department of Pathology and Laboratory Medicine, Memorial Sloan Kettering Cancer Center, New York, NY 10065, USA; tickoos@mskcc.org (S.K.T.); reuterv@mskcc.org (V.E.R.)

**Keywords:** KRAS, papillary renal neoplasm with reverse polarity, renal cell carcinoma

## Abstract

Background/Objectives: *KRAS* mutations in renal cell carcinoma (RCC) are uncommon and most frequently described in papillary renal neoplasm with reverse polarity (PRNRP). Beyond this entity, the broader clinicopathologic and molecular features of *KRAS*-mutated RCC remain insufficiently characterized. This study aimed to provide a descriptive assessment of *KRAS*-mutated RCC. Methods: *KRAS*-mutant RCC patients were identified from the Memorial Sloan Kettering-Integrated Mutation Profiling of Actionable Cancer Targets (MSK-IMPACT) and The Cancer Genome Atlas Kidney Renal Papillary Cell Carcinoma (TCGA-KIRP) cohorts. Copy-number alterations were evaluated using Fraction and allele-specific copy number estimates from tumor sequencing (FACETS). Available samples were used for immunohistochemistry and RNA-sequencing analysis. Results: Seventeen patients were included. Three distinct *KRAS*-mutant RCC subtypes were identified: *KRAS*-mutant PRCC (35%), *KRAS*-mutant URCC (35%), and PRNRP (29%). Seven patients (41%) had metastatic disease; none were PRNRP. RNA-based deconvolution analysis revealed that PRNRP had enrichment in distal nephron components, whereas *KRAS*-mutant PRCC was enriched in proximal tubule cells (*p* = 0.02). IHC staining of L1CAM was positive in PRNRP but negative in *KRAS*-mutant PRCC, supporting their distinct cell-of-origin phenotypes. This study is limited by its cohort size, which influences the availability of tissue samples. Conclusions: PRNRP represents a distinct *KRAS*-mutant RCC subtype with unique metabolic and genomic features linked to its distal nephron origin. This contrasts with the genomic complexity and aggressive clinical behavior observed in *KRAS*-mutant PRCC and URCC, highlighting the need for subtype-specific diagnostic criteria and therapeutic strategies.

## Simple Summary

This study characterizes the clinical and molecular features of *KRAS*-mutated Renal Cell Carcinoma (RCC), identifying three distinct subtypes, which arise from different cells of origin and exhibit diverse genomic alterations and metabolic profiles. Clinical outcomes varied among *KRAS*-mutated subtypes, with 7 cases developing metastatic disease and resistance to current treatment strategies, highlighting the need for subtype-specific diagnostic criteria and therapeutic strategies.

## 1. Introduction

The *KRAS* oncogene is one of the most commonly altered genes in human solid tumors, with activating mutations detected in up to 80% of pancreatic cancers and a substantial proportion of colorectal and lung cancers [1,2]. These mutations lead to continuous activation of the RAS–MAPK signaling pathway, which drives tumor initiation and progression, though their impact on prognosis varies by cancer type. Recent advances in the development of mutation-specific *KRAS* inhibitors have renewed interest in understanding the impact of *KRAS* mutations across different malignancies, emphasizing the need for precise characterization of *KRAS*-driven tumor biology to guide targeted therapies [2,3].

*KRAS* mutations in renal cell carcinoma (RCC) are rare, occurring in 1% of cases [4,5,6,7]. Recent efforts have identified an association with a subtype of papillary RCC known as “papillary renal neoplasm with reverse polarity” (PRNRP), which has a distinct morphology and immunohistochemical (IHC) profile [4,5,7,8,9]. PRNRP tumors are often small (<4.5 cm), localized lesions, and are considered to have a favorable prognosis [8,9,10,11,12]. However, the impact of *KRAS* alterations on oncogenesis is tissue-specific, and their role in RCC remains poorly understood [1,13].

While *KRAS* mutations are often found in PRNRP, other genomic alterations appear to be heterogeneous [5,8,11,14]. Furthermore, the prevalence and significance of *KRAS* mutations across the broader spectrum of RCC remain unclear. With the emergence of targeted therapies for *KRAS* mutations, there is a need to better characterize *KRAS*-mutant RCC and its clinical outcomes [15].

A retrospective clinical and genomic database was utilized to investigate the characteristics associated with *KRAS*-mutant RCC. For available tumor samples, RNA sequencing and IHC were performed to evaluate phenotypic patterns. We sought to assess the impact of *KRAS* mutations on RCC histology and clinical outcomes.

## 2. Materials and Methods

### 2.1. Cohort Selection

All patients with *KRAS*-mutant RCC were identified within the Memorial Sloan Kettering-Integrated Mutation Profiling of Actionable Cancer Targets (MSK-IMPACT) cohort between 2006 and 2023. The cohort included targeted next-generation sequencing (NGS) panels used to profile more than 129 cancer-associated genes in tumor samples from patients as part of their routine clinical care [16]. Institutional review board approval was obtained, and all patients provided informed consent for genomic sequencing and data collection. All methods were performed in accordance with the institutional guidelines and regulations.

Patients were excluded if (1) the *KRAS* mutation was not identified as an oncogenic driver according to the OncoKB database (*N* = 1); (2) clinical records were unavailable (*N* = 1); or (3) they were diagnosed with a histological subtype known to exhibit distinct biological features and driver mutations, such as pediatric metanephric adenoma (*N* = 1) and clear cell renal cell carcinoma (*N* = 2) [17]. Only RCC subtypes in which *KRAS* mutations could influence oncogenesis were included.

Clinical data were obtained through manual review of institutional electronic medical records at Memorial Sloan Kettering Cancer Center. A descriptive analysis was performed to evaluate patient and tumor characteristics, disease presentation, progression patterns, and differences in management strategies. Aggressive tumor behavior was defined by the presence or development of metastasis.

In addition, *KRAS*-mutant RCC cases from The Cancer Genome Atlas Kidney Renal Papillary Cell Carcinoma (TCGA-KIRP) repository that met the inclusion criteria were added to the analysis (https://portal.gdc.cancer.gov/projects/TCGA-KIRP accessed on 23 November 2024). Relevant clinical data were then extracted from cBioPortal [18,19,20].

### 2.2. Pathologic Evaluation

All *KRAS*-mutant RCC cases were retrospectively reviewed by a specialized genitourinary pathologist (YC) to ensure consistent classification on the basis of the World Health Organization (WHO, 5th ed) diagnostic criteria [21]. The digital pathologic images from the TCGA-KIRP repository cases were reviewed by the same pathologist to ensure consistency.

### 2.3. Genomic Characterization and Analysis

All MSK-IMPACT study patients had undergone targeted DNA next-generation sequencing of tumor samples from the primary tumor, except for two patients whose samples were taken from metastatic sites [16]. The mutational profile of the cohort was characterized.

Fraction and allele-specific copy number estimates from tumor sequencing (FACETS) were performed on 15 cases to obtain arm- and gene-level copy number (CN) alterations through total copy number (TCN), major copy number (MCN) and minor copy number (LCN) values [22]. CN status was defined as follows: tumors with no alterations were classified as diploid, CN gains were defined by a TCN ≥ 2, amplifications were defined as a TCN ≥ 5, and CN losses were defined as an LCN of 0. Tumor content and *KRAS* clonality on MSK-IMPACT were estimated using FACETS. A mutation was classified as clonal if the cancer cell fraction value obtained from FACETS was greater than 80%, otherwise the mutation was classified as sub-clonal. For four samples, purity and clonality were indeterminate by FACETS, however they were included in the analysis.

### 2.4. Transcriptomic Characterization and Analysis

Four institutional primary tumors were available for transcriptional profiling. In addition, RNA sequencing data for the TCGA-KIRP samples were downloaded from the National Institutes of Health (NIH) Genomic Data Commons [23]. The tumor samples were classified on the basis of their histological subtype: four PRNRP samples, four *KRAS*-mutant PRCC samples, and one *KRAS*-mutant URCC sample.

RNA sequencing reads were aligned against human genome assembly hg19 by STAR 2-pass alignment [24]. QC metrics, for example, general sequencing statistics, gene feature and body coverage, were then calculated based on the alignment result through RSeQC [25]. RNA sequencing gene-level count values were computed by using the R package GenomicAlignments version 1.14.2 over aligned reads with UCSC KnownGene in hg19 as the base gene model [26,27]. The Union counting mode was used and only mapped paired reads after alignment quality filtering were considered. Finally, gene level FPKM (Fragments Per Kilobase Million) and raw read count values were computed by the R package DESeq2 [28].

Principal component analysis (PCA) and differential expression of genes (DEGs) were performed via the R package DESeq2 [28]. For the DEGs, only the PRNRP and *KRAS*-mutant PRCC cases were assessed. *KRAS*-mutant URCC patients were not included in this analysis. Genes with a log2-fold change > 1 and an adjusted *p* value < 0.05 were considered differentially expressed.

The DEG analysis results were used as inputs for gene set enrichment analyses (GSEAs) against three gene set collections: hallmark, GO:BP (C5), and canonical pathways (CP) from MSigDB through the R package clusterProfiler [29,30,31,32,33].

The single-sample GSEA method implemented in the R GSVA package was employed for cell of origin and immune deconvolution analysis [34].

### 2.5. Immunohistochemistry Evaluation

Immunohistochemistry was performed on all of the institutional PRNRP and *KRAS*-mutant PRCC cases, as well as on four of the six available *KRAS*-mutant URCC cases, utilizing 5-µm-thick formalin-fixed, paraffin-embedded tissue sections. The sections were stained on the BenchMark ULTRA automated immunostainer (Roche) using L1CAM antibody (14.10, Biolegend, 1:400). L1CAM is a marker for the collecting duct and connecting tubule principal cells [35]. The staining was scored from 0 (no staining) to 3+ (strong staining). A sample was considered positive if it showed moderate to strong staining (≥2+) in at least 10% of tumor cells.

## 3. Results

### 3.1. Clinicopathologic Features

A total of 1790 patients with RCC were identified between 2006 and 2023 within the MSK-IMPACT cohort, of whom 16 patients (0.9%) had a mutation within the *KRAS* gene. Additionally, among 292 patients from the TCGA-KIRP dataset, 5 patients (1.8%) were found to have a *KRAS* mutation. A total of 17 patients ultimately met the inclusion criteria for the analysis.

**Figure 1:**
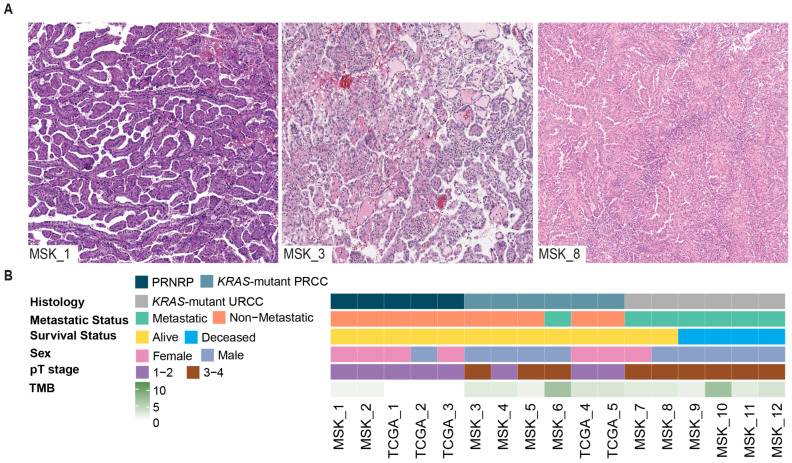
Clinical Overview of *KRAS*-Mutant Renal Cell Carcinoma (RCC) Across Individual Patient Tumors. (**A**) Representative H&E images of *KRAS*-mutant RCC: papillary renal neoplasm with reverse polarity (MSK_1), *KRAS*-mutant papillary RCC (MSK_3), and *KRAS*-mutant unclassified RCC (MSK_8). (**B**) Clinical Data Overview. TMB: tumor mutation burden.

The median age at diagnosis was 61 years (IQR = 46, 66), and 11 patients were men (65%). Patients had a median primary tumor size of 4.2 cm (IQR = 2.7, 7.4), with the majority presenting with pT3 and pT4 stages (*N* = 5, 29% and *N* = 4, 24%, respectively). Three distinct histologic subtypes were identified: *KRAS*-mutant PRCC (*N* = 6, 35%), *KRAS*-mutant URCC (*N* = 6, 35%), and PRNRP (*N* = 5, 29%). The *KRAS*-mutant URCC group presented mainly high-grade tubulopapillary, poorly differentiated, or sarcomatoid morphologies and lacked areas compatible with classic PRCC. A representative image of each subtype is presented in Figure 1A. The median follow-up period was 35 months (IQR = 10.7, 74.2).

Thirteen patients (76%) presented with localized disease and underwent upfront surgical resection. Four patients (24%) had metastatic disease at presentation, all with unclassified histology. Additionally, two *KRAS*-mutant URCCs and one *KRAS*-mutant PRCC developed metastases during the follow-up period, resulting in a total of 7 patients (41%) with metastatic disease. None of the PRNRP patients experienced recurrence or metastasis.

All patients who initially presented with metastatic disease received systemic therapy. Patients who progressed to metastatic disease after initial nephrectomy were treated with tyrosine kinase inhibitors, and all experienced disease progression. Four patients with metastatic disease died (24%) (Supplementary Figure S1).

### 3.2. Genomic Characterization and Analysis

Sixteen patients (94%) had a *KRAS*-activating missense mutation, and one patient (6%) with unclassified histology had an in-frame insertion mutation. The predominant *KRAS* mutations identified were G12D (*N* = 9, 53%) and G12V (*N* = 5, 29%). Notably, all of the identified missense mutations have been previously reported in prior PRNRP cohorts [4,5,8,11,14]. The *KRAS* mutation was clonal in 12 out of the 13 patients (92%). All patients had a low TMB, with a median of 2.6 mutations per million bases of coding DNA (IQR = 1.6, 3.1).

**Figure 2:**
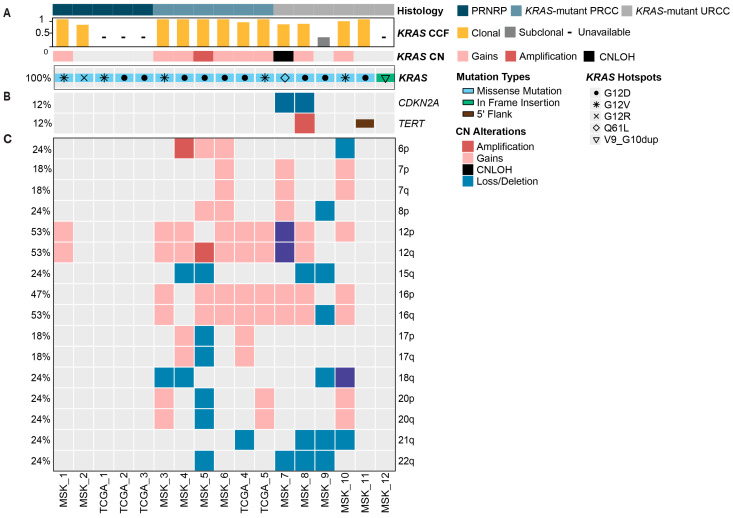
Genomic Overview of *KRAS*-Mutant Renal Cell Carcinoma (RCC) Across Individual Patient Tumors. (**A**) *KRAS* mutation characterization. (**B**) Co-mutations/Gene-level CN alterations present with a frequency of ≥12%. (**C**) Arm-level copy number alterations with a frequency of ≥18%. The alteration frequency (%) is listed on the left. CCF: cancer cell fraction, CN: copy number, CNLOH: copy-neutral loss of heterozygosity.

At the *KRAS* gene level, the *KRAS*-mutant PRCC presented either gain (*N* = 5) or amplification (*N* = 1). In PRNRP, only one patient had a gain in *KRAS* copy number (CN). Additionally, 1 *KRAS*-mutant URCC had copy-neutral loss of heterozygosity (CN-LOH), with both LOH and a TCN greater than 2 in the *KRAS* gene (Figure 2A).

Overall, the PRNRP patients presented no chromosomal CN alterations, only one had a chromosome 12 gain, which is consistent with previous PRNRP cohorts [14]. In comparison, the *KRAS*-mutant PRCC cases frequently harbored gains of chromosomes 12 (*N* = 5) and 16 (*N* = 4). Although this spectrum overlaps with that of classic PRCC, the most frequent gain of chromosome 12 in *KRAS*-mutant PRCC differs from that in classic PRCC and coincides with the *KRAS* gene locus (12p12.1) (Supplementary Figure S2) [9,14]. The *KRAS*-mutant URCC exhibited a more variable pattern of CN alterations, with gains on chromosomes 7 (*N* = 2), 12 (*N* = 2) and 16 (*N* = 3) and loss of chromosomes 21 (*N* = 3) and 22 (*N* = 3) (Figure 2C).

Clinically aggressive tumors presented a greater frequency of altered genes, including *TERT* mutations and amplifications, as well as *CDKN2A* deletions. Both were present in two patients (12%) (Figure 2B) and have been previously associated with poorer prognosis in RCC [9,36,37]. Additional co-mutations found in this group included *NF2*, *B2M*, and *SETD2*, each present in only 1 case (6%). All OncoKB-altered genes are presented in Supplementary Figure S3.

### 3.3. Transcriptomic Characterization and Analysis

Principal component analysis (PCA) revealed that the PRNRP samples grouped together and were separate from the *KRAS*-mutant PRCC or *KRAS*-mutant URCC samples. There was no evidence of a batch effect after integration, although the *KRAS*-mutant PRCC samples were distinguishable from their original cohort (Supplementary Figure S4).

**Figure 3:**
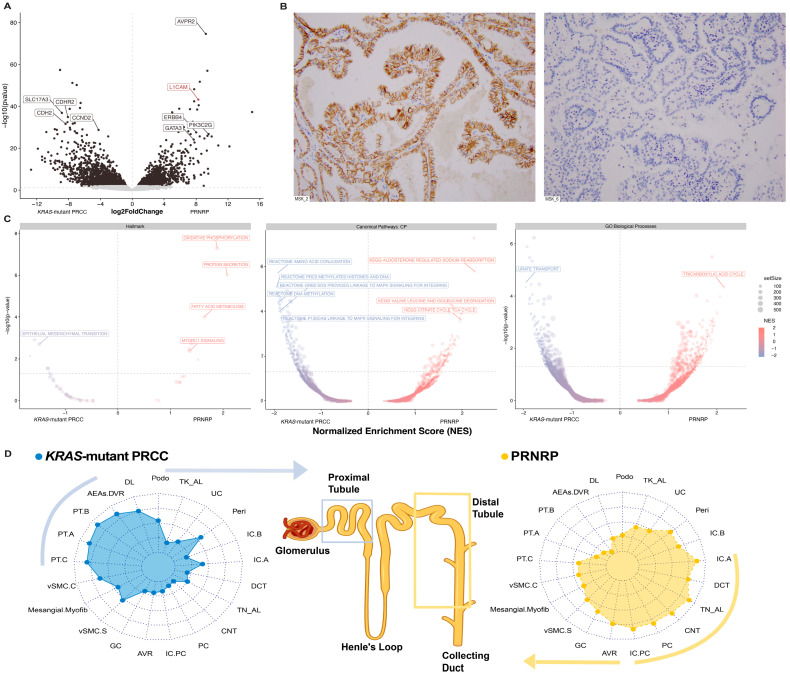
Transcriptomic overview of *KRAS*-mutant PRCC and PRNRP. (**A**) Differential gene expression in *KRAS*-mutant PRCC and PRNRP. (**B**) Representative L1CAM-positive PRNRP (MSK_2) and L1CAM-negative *KRAS*-mutant PRCC (MSK_6) samples. (**C**) Gene set enrichment analysis of *KRAS*-Mutant PRCC and PRNRP via three distinct gene set collections. For each enriched gene set, the normalized enrichment score (NES) and gene set size (number of genes) are indicated. (**D**). Cell type signature average scores for PRNRP (*N* = 4) and *KRAS*-mutant PRCC (*N* = 4). UC: uncharacterized endothelial cells; Peri: pericytes; IC.B: Intercalated cell type B; IC.C: Intercalated cell type C; DCT: distal convoluted tubule; TN_AL: thin ascending limb; CNT: connecting tubule; PC: principal cells; IC.PC: intercalated/principal cell hybrid; AVR: ascending vasa recta; GC: glomerular capillaries; vSMC.S: vascular smooth muscle cell synthetic phenotype; Mesangial.Myofibs: mesangial cells; vSMCs.C: vascular smooth muscle cell contractile phenotype; PT.C: Proximal tubule cell C; PT.A: Proximal tubule cell A; PT.B: Proximal tubule cell B; AEAs.DVR: afferent/efferent arterioles/descending vasa recta; DL: descending limb; Podo: podocyte; TK_AL: thick ascending limb.

DEG analysis revealed genes that were differentially expressed between PRNRP and *KRAS*-mutant PRCC (Figure 3A). PRNRP expressed L1CAM. Hallmark GSEA identified eight enriched biological processes in PRNRP, including “Oxidative Phosphorylation”, “Fatty Acid Metabolism”, “Apical Surface” and “MTORC1 Signaling”. In contrast, the genes associated with the *KRAS*-mutant PRCC were enriched in four processes, notably “epithelial-mesenchymal transition” and “hypoxia”. CP GSEA further highlighted distinct pathway enrichments between the two groups. A key finding in PRNRP was the enrichment of “KEGG aldosterone-regulated sodium reabsorption”, which is consistent with the V2 receptor expressed in the distal convoluted tubules and collecting ducts (Figure 3C) [38].

The cell of origin deconvolution analysis indicated that the PRNRP group was enriched in distal convoluted and connecting tubule signatures (*p*-adjusted = 0.02). In contrast, the *KRAS*-mutant PRCC samples had greater representation of the proximal tubule (PT) (*p*-adjusted = 0.02) and descending limb (*p*-adjusted = 0.02) (Figure 3D). The same analysis was performed for the *KRAS*-mutant URCC case, which had higher expression of PT cells (Supplementary Figure S5).

Finally, immune deconvolution analysis revealed that the PRNRP had increased expression of “Angiogenesis” and “Programmed Death-Ligand 1”. However, the results were not statistically significant.

### 3.4. Immunohistochemistry Correlation

L1CAM staining of the PRNRP samples revealed diffuse positive staining. In contrast, all the *KRAS*-mutant PRCC samples stained negatively. A representative case of each subtype is shown in Figure 3B. The results of the *KRAS*-mutant URCC cases were variable: two cases were negative, one was 2+, and the remaining one was 3+.

## 4. Discussion

*KRAS* mutations in RCC are infrequently reported and are most often associated with localized, indolent tumors exhibiting papillary renal neoplasm with reverse polarity (PRNRP) morphology [4,5,6,8,9,11,12]. The recent WHO classification has included PRNRP as a papillary RCC (PRCC) subtype, recognizing its unique morphologic features and indolent clinical course [9]. However, comprehensive molecular data on *KRAS*-mutated RCC remain limited, and the extent to which PRNRP differs biologically and clinically from other *KRAS*-mutated RCC types is not well established.

Our study provides a comprehensive, descriptive characterization of *KRAS*-mutant RCC, integrating clinicopathologic, genomic, and transcriptomic features. PRNRP patients within our cohort demonstrated unique genomic stability with minimal *KRAS* gene and chromosomal copy number (CN) alterations and co-mutations. This contrasts with the *KRAS*-mutant PRCC and URCC cases, which presented *KRAS* gains along with chromosomal CN alterations and co-mutations. The genomic profile of PRNRP suggests fundamental biological differences that contribute to its indolent nature.

Our findings also highlight the rarity and clinical heterogeneity of *KRAS*-mutant RCC. While PRNRP was exclusively observed in localized cases, metastatic progression was observed in *KRAS*-mutant URCC and in one *KRAS*-mutant PRCC case. The presence of additional co-mutations in these patients suggests a role for cooperative genetic alterations in driving aggressive disease behavior [13]. Neither *KRAS* mutation subtype nor *KRAS* or chromosome 12 CN alterations were associated with metastatic progression or therapy response in this cohort. While chromosome 12 CN alterations are common in *KRAS*-mutant pancreatic and colorectal cancers and linked to allelic imbalance and poorer outcomes, this association was not observed in this study [2,39].

To understand the phenotypic patterns and clinical outcomes observed, we integrated transcriptomic data to assess whether variations in the cells of origin and tissue microenvironment influenced *KRAS*-mediated tumor behavior [13,15,40]. DEGs revealed enrichment in the AVPR2 gene for PRNRP, a receptor that is expressed by the distal convoluted tubule and collecting duct [38]. L1CAM was also enriched, and the results of IHC support our findings, with L1CAM being positive in all PRNRP patients. In contrast, the *KRAS*-mutant PRCC group was negative for L1CAM and showed enrichment of CDH6 and SLC17A3, both of which are associated with proximal tubule cells [38].

The DEG and GSEA results suggest that tumor metabolism and the *KRAS* signaling pathway are different between PRNRP and *KRAS*-mutant PRCC. PRNRP strongly expressed genes that are part of the MTORC1 signaling pathway; moreover, these tumors are more metabolically active, with enrichments in oxidative phosphorylation and the TCA cycle. Additionally, its indolent clinical course can be associated with the upregulation of apical surface pathways, which function as tumor suppressors [41,42].

In contrast, *KRAS*-mutant PRCC tumors presented increased hypoxia signaling, suggesting that a different metabolic pathway is involved, which is consistent with the Warburg effect and EMT enrichment [1,43]. Additionally, this subgroup has upregulated DNA methylation pathways linked to MAPK signaling, as previously described in *KRAS*-mutant pancreatic cancer under EMT and hypoxic conditions [43]. Furthermore, the metastatic potential of *KRAS*-mutant PRCC is supported by its occurrence in one patient. The limited transcriptomic data we obtained from one *KRAS*-mutant URCC patient suggest that a subset of cases in this heterogeneous group may be related to *KRAS*-mutant PRCC, given the overlapping chromosomal abnormalities and similar cells of origin.

Our findings also suggest that PRNRP originates from cells of the distal nephron, whereas *KRAS*-mutant PRCC may be derived from proximal tubule cells. However, owing to the small cohort size, a definitive cell of origin could not be determined.

Nevertheless, accumulating evidence supports re-evaluating the classification and treatment approach of PRNRP. This includes separating PRNRP into a biologically and clinically distinct entity from *KRAS*-mutant PRCC, and accordingly, developing tailored management strategies for each subtype within the evolving molecularly defined renal cell carcinomas [9,44].

These findings are consistent with those presented by Zhang et al., who identified distinct cells of origin among two *KRAS*-mutant types, although specific histologic characterization was not presented [15]. Additionally, Tong et al. reported that PRNRP shares a similar mRNA expression profile with the distal nephron tubule, reinforcing the hypothesis of its distinct biological origin [4]. More recently, Antic et al. identified three RCC subsets with RAS-pathway alterations. One of their groups which is characterized by having metastatic potential, additional co-mutations, and CN alterations, is consistent with our *KRAS*-mutant PRCC subgroup; whereas PRNRP was classified as a separate indolent group, reinforcing its separation as a biologically and clinically distinct entity [7].

The metastatic *KRAS*-mutant RCC patients in our cohort experienced disease progression regardless of the systemic agent used. The presence of *KRAS* mutations suggests that *KRAS* inhibitors could offer new treatment options for this subgroup of patients with advanced RCC [45,46]. However, there is currently a lack of evidence supporting its efficacy in RCC. Moreover, the presence of EMT in *KRAS*-mutant PRCC may limit its potential effectiveness, as it is a known intrinsic resistance mechanism in *KRAS*-mutant lung cancer [46].

This study has several limitations. First, given the low prevalence of *KRAS* mutations in RCC, our cohort size was small, which limits the availability of tissue samples and restricts the generalizability of the results. Second, the lack of prospective experimental validation restricts conclusions to identifying associations. Nevertheless, comprehensive characterization through gene expression profiling, pathway enrichment analysis, and cell signatures provides robust evidence to support the distinct patterns identified between the *KRAS*-mutant RCC subtypes. Additionally, our unique clinical cohort allows further validation of the clinical significance of these molecular subtypes, which may have future implications for diagnosis and treatment selection.

## 5. Conclusions

PRNRP represents a distinct *KRAS*-mutant RCC subtype with unique metabolic and genomic features linked to its distal nephron origin. This contrasts with the genomic complexity and aggressive clinical behavior seen in *KRAS*-mutant PRCC and URCC, establishing PRNRP as a biologically and clinically distinct entity within the spectrum of *KRAS*-mutant RCC. These results highlight the importance of developing subtype-specific diagnostic criteria and management strategies to prevent overtreatment of this indolent tumor type and offer a molecular framework for the potential application of *KRAS*-targeted therapies in selected cases.

## Data Availability

The datasets generated and/or analyzed during the current study are available in from the National Institutes of Health (NIH) Genomic Data Commons (https://portal.gdc.cancer.gov/projects/TCGA-KIRP accessed on 23 November 2024) and Gene Expression Omnibus (GEO) repository (GSE309610). The TCGA-KIRP sample IDs analyzed in this study correspond to the following identifiers: TCGA_1: TCGA-2Z-A9JN, TCGA_2: TCGA-A4-831, TCGA_3: TCGA-BQ-5883, TCGA_4: TCGA-GL-A9DC, and TCGA_5: TCGA-MH-A854.

## Supplementary Materials

The following supporting information can be downloaded at: https://www.mdpi.com/article/10.3390/cancers17233832/s1, Supplementary Figure S1. Treatment Sequencing and Clinical Course Overview of Metastatic *KRAS*-Mutant RCC Patients. URCC: unclassified renal cell cancer, PRCC: papillary renal cell cancer, POD: disease progression, ICI: immune checkpoint inhibitor, TKI: tyrosine kinase inhibitor, mTORi: mammalian target of rapamycin inhibitor; Supplementary Figure S2. *KRAS*-Mutant RCC vs. Classical PRCC (cases were obtained from the MSK-IMPACT cohort from cBioPortal); Supplementary Figure S3. Clinical and Genomic Features of *KRAS*-Mutant RCC. *Excluded patients TMB: tumor mutation burden, MSI: microsatellite instability, CCF: cancer cell fraction, CN: copy number, CNLOH: copy neutral loss of heterozygosity; Supplementary Figure S4. Principal component analysis; Supplementary Figure S5. Cell type signature average scores a *KRAS*-mutant unclassified RCC (*N* = 1). UC: uncharacterized endothelial cells; Peri: pericytes; IC.B: Intercalated cell type B; IC.C: Intercalated cell type C; DCT: distal convoluted tubule; TN_AL: thin ascending limb; CNT: connecting tubule; PC: principal cells; IC.PC: intercalated/principal cell hybrid; AVR: ascending vasa recta; GC: glomerular capillaries; vSMC.S: vascular smooth muscle cell synthetic phenotype; Mesangial.Myofibs: mesangial cells; vSMCs.C: vascular smooth muscle cell contractile phenotype; PT.C: Proximal tubule cell C; PT.A: Proximal tubule cell A; PT.B: Proximal tubule cell B; AEAs.DVR: afferent/efferent arterioles/descending vasa recta; DL: descending limb; Podo: podocyte; TK_AL: thick ascending limb.
