# Supplementary material for: Clinical and Molecular Characterization of KRAS-Mutated Renal Cell Carcinoma
Source: Cancers (Basel). 2025 Nov 29;17(23):3832. doi: 10.3390/cancers17233832 (PMC12691090; doi:10.3390/cancers17233832)
Supplement: Supplementary file 1 [file cancers-17-03832-s001.zip › Supplementary.pdf]

Supplementary

Supplementary Figures

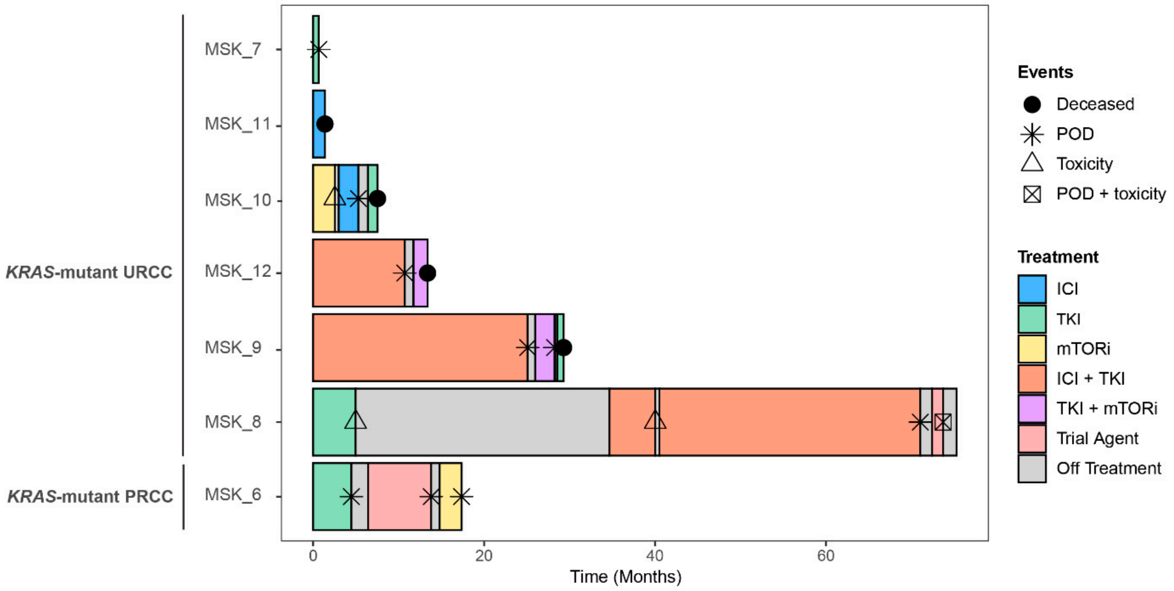

Supplementary Figure S1. Treatment Sequencing and Clinical Course Overview of Metastatic *KRAS*-Mutant RCC Patients. URCC: unclassified renal cell cancer, PRCC: papillary renal cell cancer, POD: disease progression, ICI: immune checkpoint inhibitor, TKI: tyrosine kinase inhibitor, mTORi: mammalian target of rapamycin inhibitor.

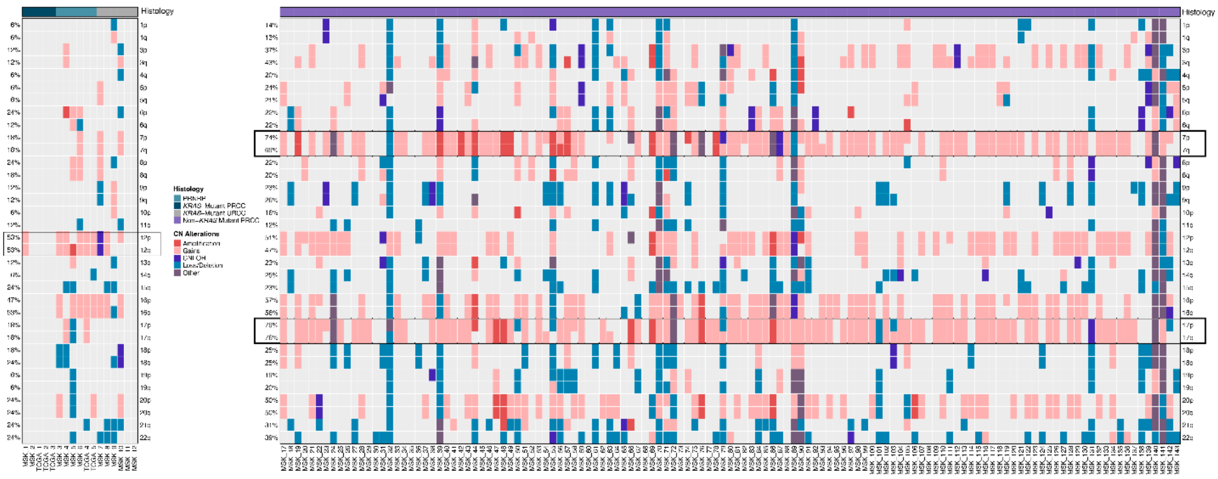

Supplementary Figure S2. *KRAS*-Mutant RCC vs Classical PRCC (cases were obtained from the MSK-IMPACT cohort from cBioPortal).

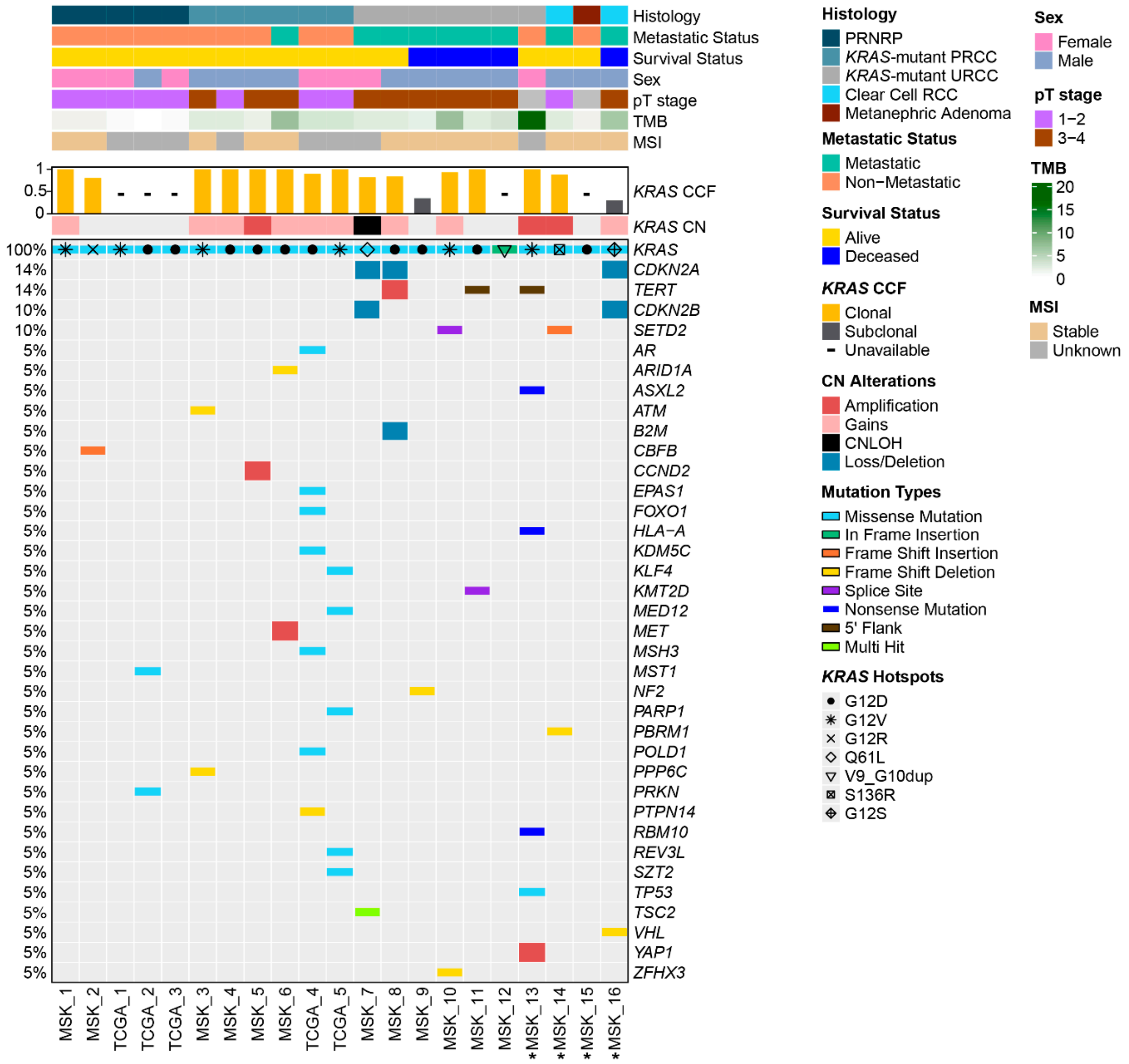

Supplementary Figure S3. Clinical and Genomic Features of *KRAS*-Mutant RCC.

*Excluded patients

TMB: tumor mutation burden, MSI: microsatellite instability, CCF: cancer cell fraction, CN: copy number, CNLOH: copy neutral loss of heterozygosity.

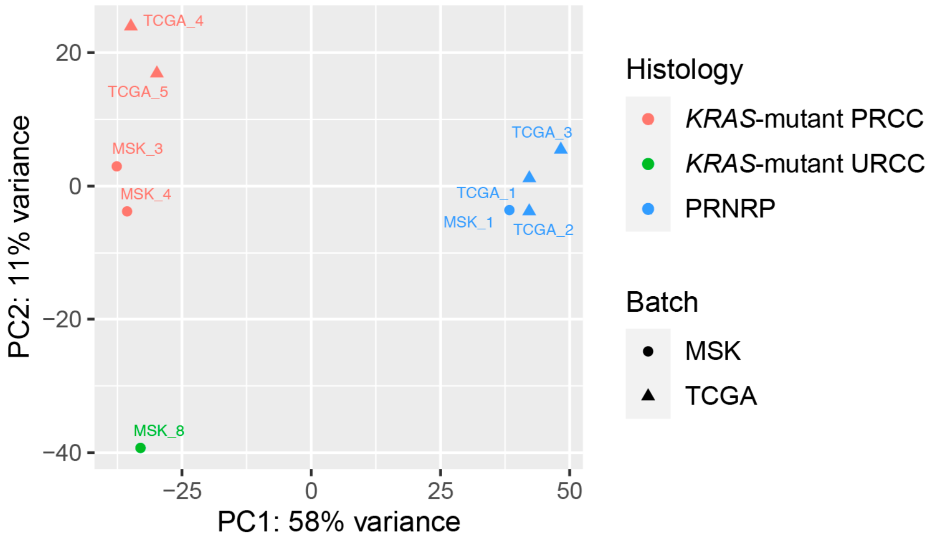

Supplementary Figure S4. Principal component analysis.

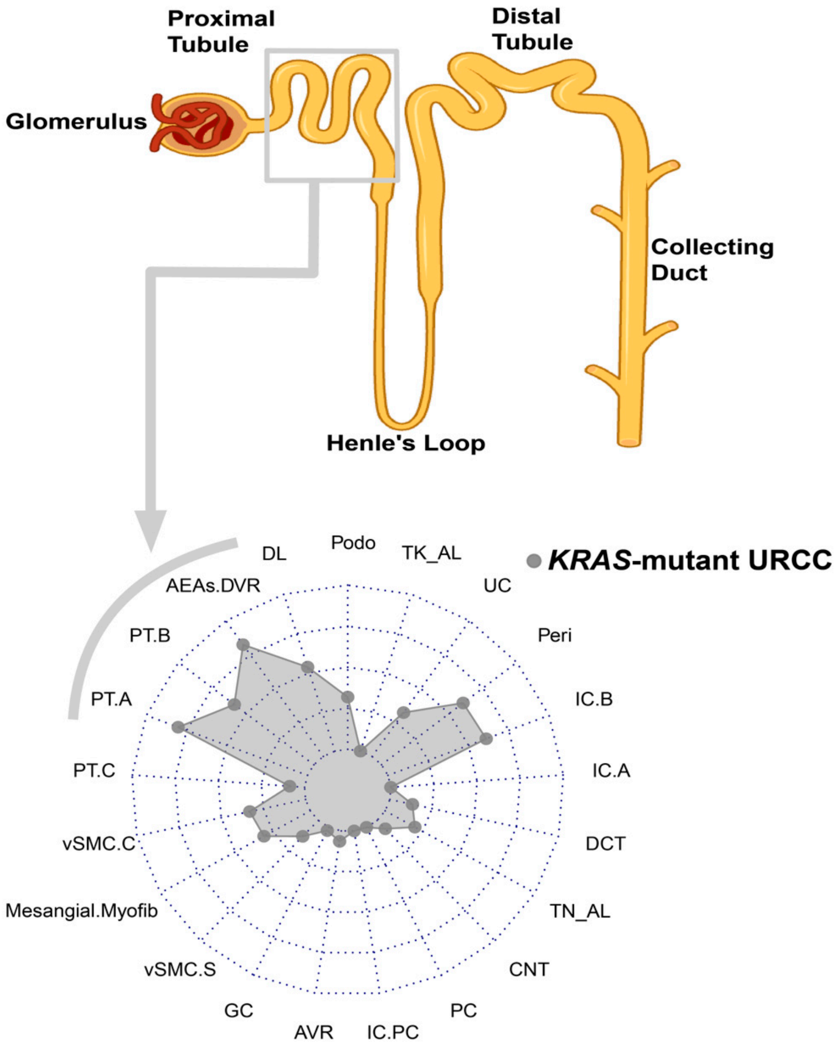

Supplementary Figure S5. Cell type signature average scores of a *KRAS*-mutant unclassified RCC (N=1).

UC: uncharacterized endothelial cells; Peri: pericytes; IC.B: Intercalated cell type B; IC.C: Intercalated cell type C; DCT: distal convoluted tubule; TN_AL: thin ascending limb; CNT: connecting tubule; PC: principal cells; IC.PC: intercalated/principal cell hybrid; AVR: ascending vasa recta; GC: glomerular capillaries; vSMC.S: vascular smooth muscle cell synthetic phenotype; Mesangial.Myofibs: mesangial cells; vSMCs.C: vascular smooth muscle cell contractile phenotype; PT.C: Proximal tubule cell C; PT.A: Proximal tubule cell A; PT.B: Proximal tubule cell B; AEAs.DVR: afferent/efferent arterioles/descending vasa recta; DL: descending limb; Podo: podocyte; TK_AL: thick ascending limb.
