# Supplementary figures and images for: Clinical and Molecular Characterization of KRAS-Mutated Renal Cell Carcinoma
Source: Cancers (Basel). 2025 Nov 29;17(23):3832. doi: 10.3390/cancers17233832 (PMC12691090; doi:10.3390/cancers17233832)

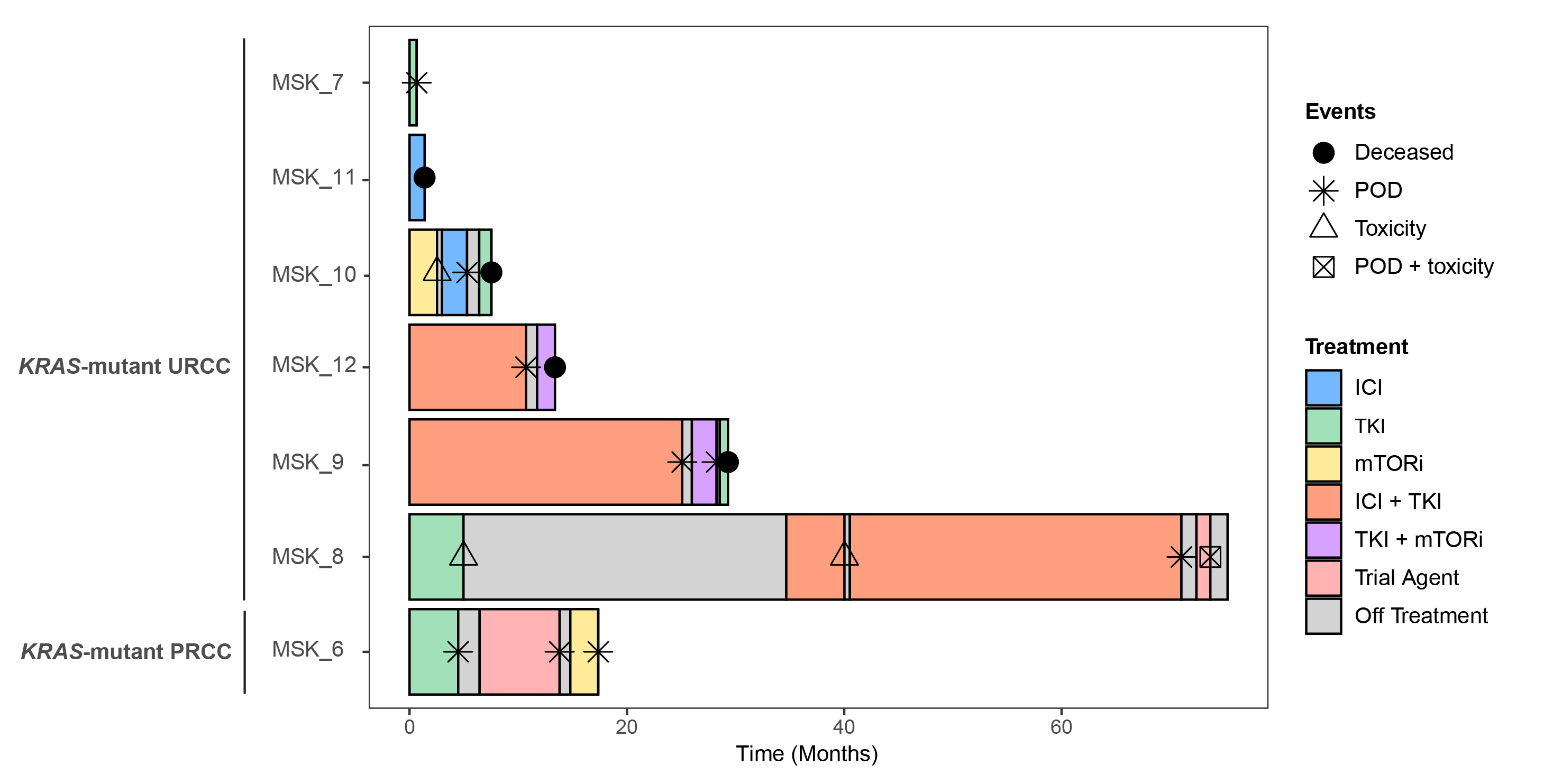

Supplement: Supplementary file 1 [file cancers-17-03832-s001.zip › S1.png]

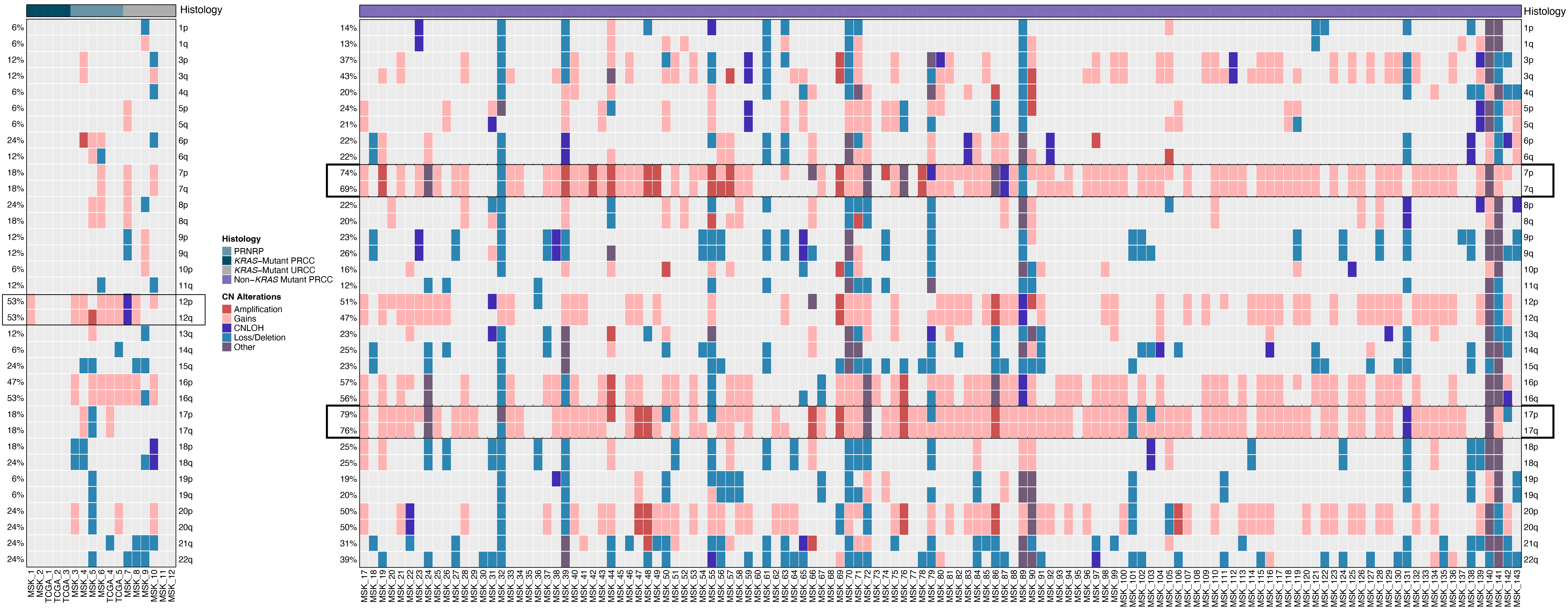

Supplement: Supplementary file 1 [file cancers-17-03832-s001.zip › S2.png]

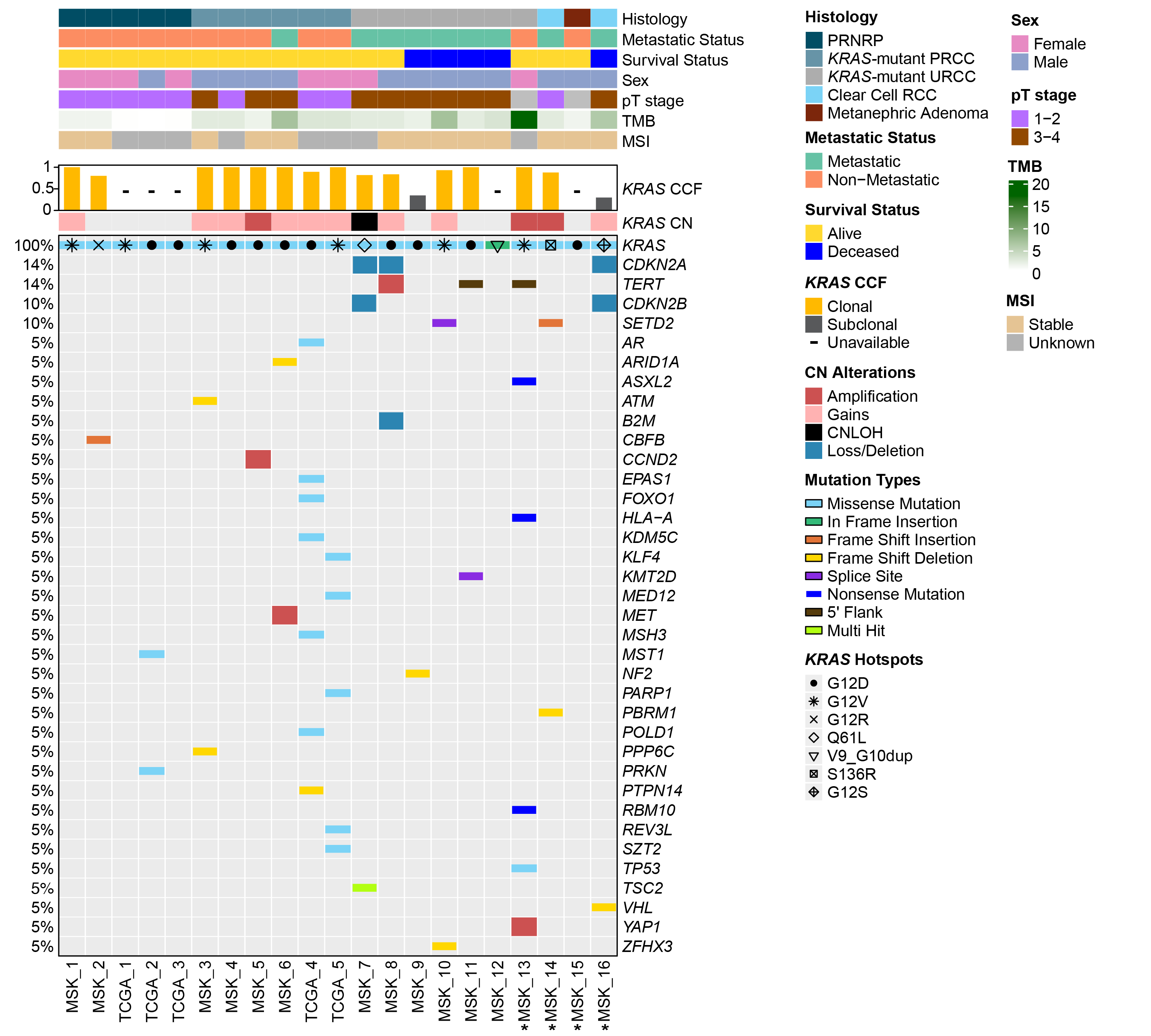

Supplement: Supplementary file 1 [file cancers-17-03832-s001.zip › S3.png]

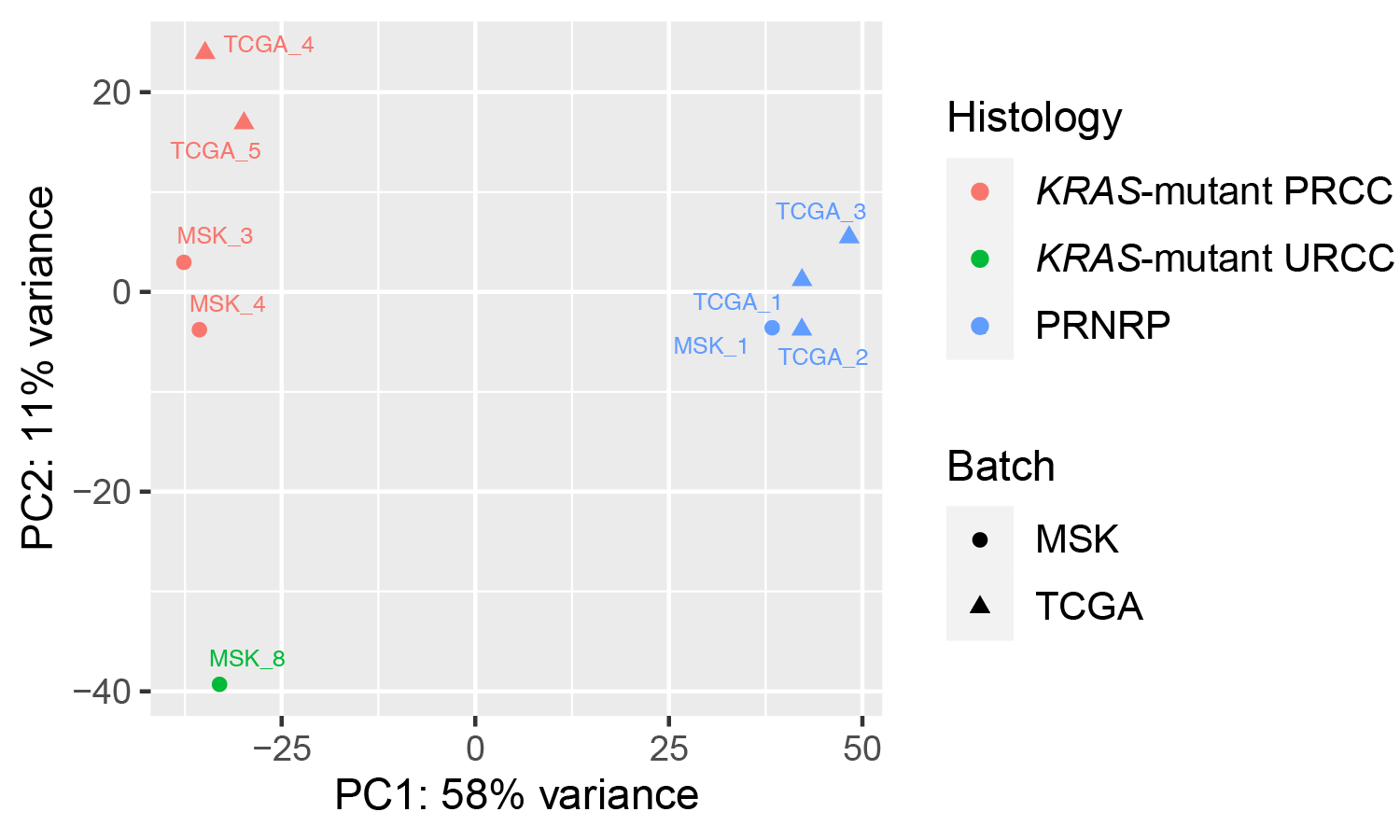

Supplement: Supplementary file 1 [file cancers-17-03832-s001.zip › S4.png]

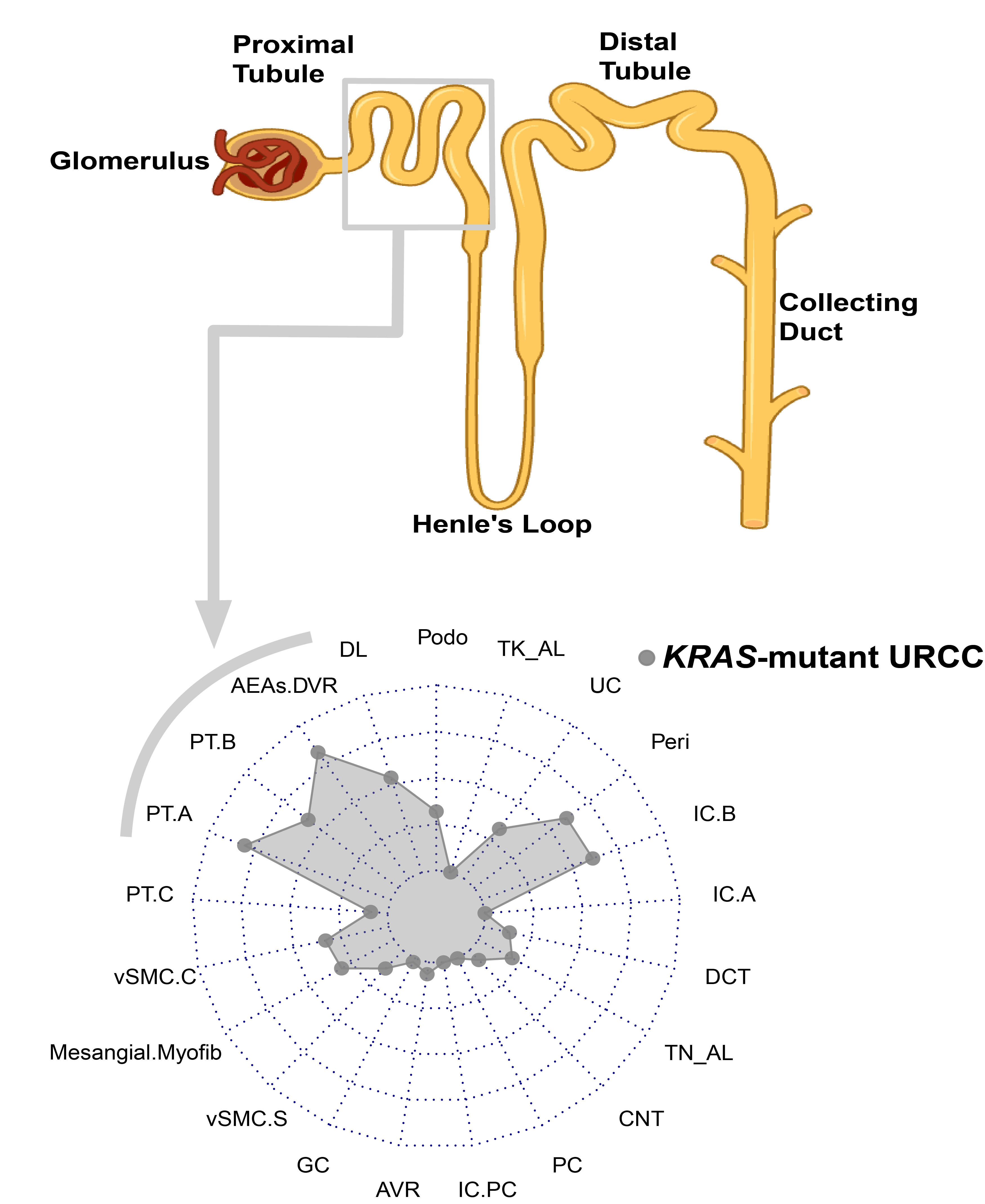

Supplement: Supplementary file 1 [file cancers-17-03832-s001.zip › S5.png]
